# Expression of Concern: Scottish Firefighters Occupational Cancer and Disease Mortality Rates: 2000-2020

**DOI:** 10.1093/occmed/kqaf032

**Published:** 2025-05-20

This is an expression of concern regarding: A A Stec, A Robinson, T A M Wolffe, E Bagkeris, Scottish Firefighters Occupational Cancer and Disease Mortality Rates: 2000-2020, *Occupational Medicine*, Volume 73, Issue 1, January 2023, Pages 42–48, https://doi.org/10.1093/occmed/kqac138

In April 2023, the journal’s Editor-in-Chief was alerted to concerns regarding the methodology used in the research and began an investigation. While the journal’s editorial team continue to consider how best to proceed, the journal is publishing this Expression of Concern to advise readers to examine the study with care in the interim.

